# The SIRT1 Deacetylase Suppresses Intestinal Tumorigenesis and Colon Cancer Growth

**DOI:** 10.1371/journal.pone.0002020

**Published:** 2008-04-16

**Authors:** Ron Firestein, Gil Blander, Shaday Michan, Philipp Oberdoerffer, Shuji Ogino, Jennifer Campbell, Anupama Bhimavarapu, Sandra Luikenhuis, Rafael de Cabo, Charles Fuchs, William C. Hahn, Leonard P. Guarente, David A. Sinclair

**Affiliations:** 1 Paul F. Glenn Laboratories for the Biological Mechanisms of Aging, Department of Pathology, Harvard Medical School, Boston, Massachusetts, United States of America; 2 Department of Biology, Massachusetts Institute of Technology, Cambridge, Massachusetts, United States of America; 3 Department of Pathology, Brigham and Women's Hospital and Harvard Medical School, Boston, Massachusetts, United States of America; 4 Department of Medical Oncology, Dana-Farber Cancer Institute, Boston, Massachusetts, United States of America; 5 Laboratory of Experimental Gerontology, National Institute on Aging, National Institutes of Health, Bethesda, Maryland, United States of America; 6 Department of Medical Oncology, Dana Farber Cancer Institute, and Broad Institute of Harvard and Massachusetts Institute of Technology, Boston, Massachusetts, United States of America; Ordway Research Institute, United States of America

## Abstract

Numerous longevity genes have been discovered in model organisms and altering their function results in prolonged lifespan. In mammals, some have speculated that any health benefits derived from manipulating these same pathways might be offset by increased cancer risk on account of their propensity to boost cell survival. The Sir2/SIRT1 family of NAD^+^-dependent deacetylases is proposed to underlie the health benefits of calorie restriction (CR), a diet that broadly suppresses cancer in mammals. Here we show that CR induces a two-fold increase SIRT1 expression in the intestine of rodents and that ectopic induction of SIRT1 in a β-catenin-driven mouse model of colon cancer significantly reduces tumor formation, proliferation, and animal morbidity in the absence of CR. We show that SIRT1 deacetylates β-catenin and suppresses its ability to activate transcription and drive cell proliferation. Moreover, SIRT1 promotes cytoplasmic localization of the otherwise nuclear-localized oncogenic form of β-catenin. Consistent with this, a significant inverse correlation was found between the presence of nuclear SIRT1 and the oncogenic form of β−catenin in 81 human colon tumor specimens analyzed. Taken together, these observations show that SIRT1 suppresses intestinal tumor formation *in vivo* and raise the prospect that therapies targeting SIRT1 may be of clinical use in β−catenin-driven malignancies.

## Introduction

Cancer is the second leading cause of age-related mortality in humans. Calorie restriction extends lifespan in all organisms tested and in mammals exerts strong tumor suppressive effects [1]. In lower eukaryotes, the *SIR2* gene is proposed to mediate the health benefits of CR [2], [3]. SIRT1, the mammalian ortholog of *SIR2*, is induced by CR in multiple tissues of mammals, and has been shown to ameliorate degenerative diseases associated with aging, such as neurodegeneration and metabolic decline [4]. While CR is known to inhibit both spontaneous and induced tumor formation, a role for SIRT1 in this process remains to be demonstrated [5]. There are conflicting data *in vitro* as to whether SIRT1 will be found to act as an oncogene or as a tumor suppressor but to date there have been no *in vivo* studies that address this question. On the one hand, SIRT1 is upregulated in tumors and cancer cells lacking the tumor suppressor gene, HIC1 [6], can inhibit apoptosis [7], [8], [9], [10] and down-regulates the expression of tumor suppressor genes [11], leading many to conclude that SIRT1 will prove to be an oncogene *in vivo*. On the other hand, SIRT1 can be pro-apoptotic [12] and anti-proliferative [13], [14], and consequently has been proposed to behave as a tumor suppressor *in vivo*. Moreover, some have argued that mammalian longevity genes that delay age-related atrophic diseases may conversely predispose humans to a higher incidence of cancer due to their anti-apoptotic function [15]. This study addresses this controversial question by testing the effects of SIRT1 on tumor formation and growth.

We chose to test the effect of SIRT1 in the APC^min/+^ model of colon cancer for a variety of reasons: it physiologically recapitulates the early events of colon cancer in humans, the mechanism of tumorigenesis is well characterized, and CR has previously been shown to reduce the rate of tumorigenesis in this model [16]. The APC^min/+^ mouse contains a germline mutation in the adenomatous polyposis coli (APC) tumor suppressor gene [17]. Somatic loss of the second allele leads to constitutive nuclear localization of β-catenin and adenoma formation [18], [19].

β-catenin is the central effector in the canonical Wnt signaling pathway that controls stem cell maintenance, development and carcinogenesis [20]. Constitutive activation of the β-catenin pathway has been found in 90% of colorectal cancers [21], [22]. In addition, this pathway is aberrantly activated in many other cancers including prostate, breast, ovary and melanoma. Interestingly, two recent studies have shown that increased Wnt signaling is associated with accelerated aging, suggesting that an attenuation of Wnt signaling might underlie CR and be beneficial not only for treating cancer but for more broadly attenuating diseases of aging [23], [24].

We report here that SIRT1 suppresses intestinal tumorigenesis in the APC^min/+^ mouse model and inhibits colon cancer growth. We provide substantial evidence that the anti-tumorigenic effects of SIRT1 depend on its deacetylase activity and are mediated through inhibition of β-catenin. These findings identify a tumor suppressive function for SIRT1, provide mechanistic insight, and suggest a therapeutic role for SIRT1 deacetylase activators in colon cancer.

## Results

**Figure 1:**
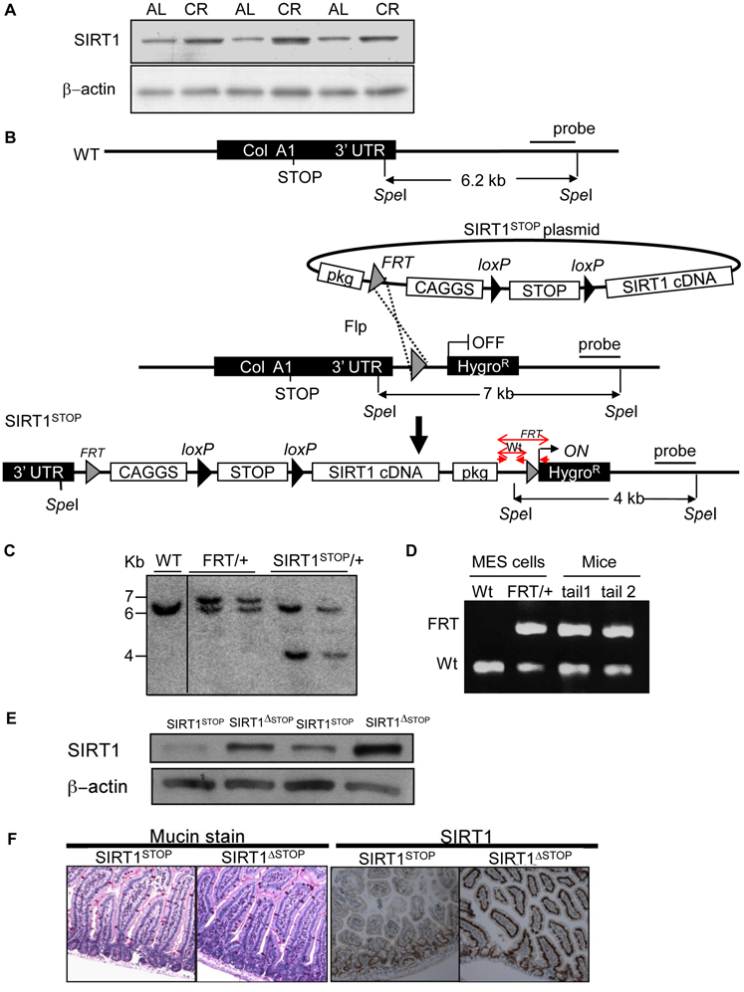
Generation of the conditional SIRT1 transgenic mice that mimic calorie restriction induced SIRT1 overexpression. (A) Western blot analysis showing expression levels in the gut epithelium of SIRT1 in ad libitum-fed (AL) or calorie restricted (CR) rats. β-actin served as the loading control in all lanes. (B) Schematic representation of the strategy used for the generation of the floxed SIRT1 mouse embryonic stem (MES) cells. SIRT1 was cloned downstream of a constitutive CAGGS promoter followed by a transcriptional loxP-STOP-loxP cassette. This construct was specifically targeted in the 3′ UTR of the collagen A1 locus (ColA1) of mouse embryonic stem cells (MES) cells by FLP recombination. The targeted MES cells were injected into blastocysts. Red arrows indicate location of the SIRT1-Tg genotyping primers. (C) Southern blot showing the confirmation of the SIRT1^STOP^ single integration into the Col1A locus of MES cells. (D) PCR confirming the germline transmission of the SIRT1^STOP^ transgene to the chimaeras' offspring. (E) Western blot showing the levels of SIRT1 in the triple transgenic mice overexpressing SIRT1 (SIRT1^ΔSTOP^) and controls (SIRT1^STOP^). β-actin served as the loading control in all lanes. (F) Mucin stain and immunohistochemistry of SIRT1 in the small intestine of experimental (SIRT1^ΔSTOP^) and controls (SIRT1^STOP^) animals.

Earlier studies have shown a dramatic tumor suppressive effect of CR but the molecular mechanism(s) are currently unknown. We observed that rats on a CR diet have ∼2-fold higher levels of SIRT1 in the gut epithelium relative to *ad lib*-fed controls (Fig. 1A). To test the effect of increasing SIRT1 expression in intestinal epithelial cells, we generated a floxed SIRT1 transgenic mouse (Fig. 1B). SIRT1 transgenic mice were crossed to APC^min/+^ mice followed by breeding to the Villin-Cre strain [25]. Thus, we generated triple transgenic mice (SIRT1^ΔSTOP^; Vil-Cre; APC^min/+^) which overexpress SIRT1 specifically in the gut villi (referred to as SIRT1^ΔSTOP^). The SIRT1 levels in the gut of SIRT1^ΔSTOP^ mice were approximately 7-fold (Fig. 1E) and the morphology of villi appeared otherwise normal (Fig. 1F).

**Figure 2:**
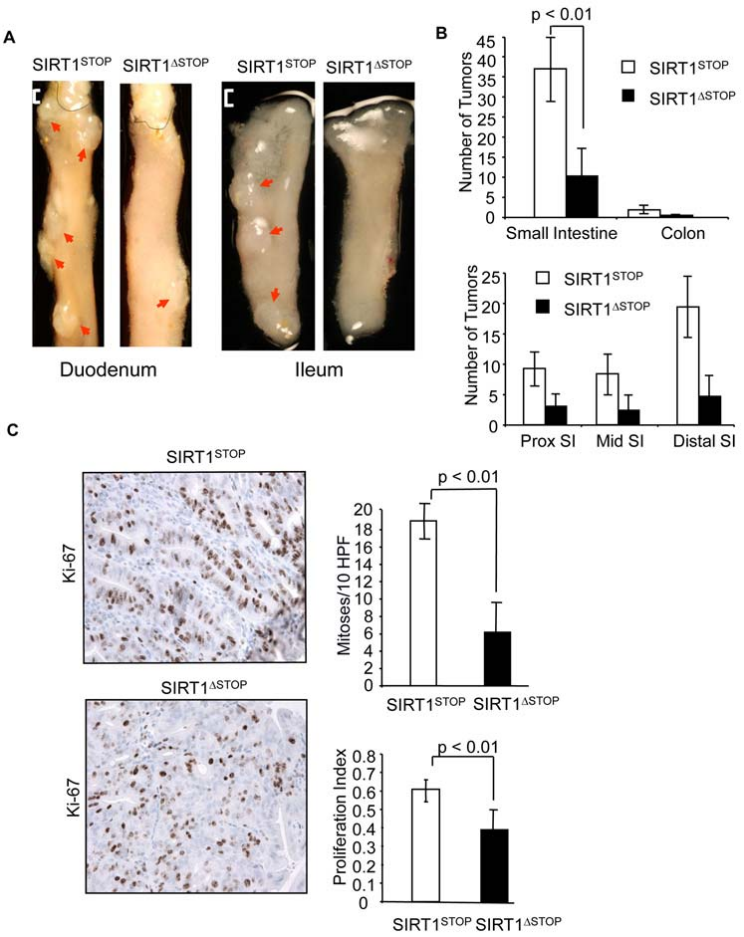
Effect of SIRT1 overexpression on intestinal tumor formation and proliferation in Apc^min/+^ mice. (A) Pictures of whole duodenal and ileal sections show gross intestinal tumors in mice overexpressing SIRT1 (SIRT1^ΔSTOP^) and controls (SIRT1^STOP^). Solid line indicates gastro-duodenal junction. Arrows indicate adenomas. White bar denotes 1 mm scale. (B) Average number of tumors according to intestinal location in SIRT1^STOP^ control (n = 8) and SIRT1^ΔSTOP^ experimental mice (n = 11). (C) Ki-67 staining of adenomas and proliferation rates. Pictures show Ki-67 immunohistochemical staining of adenomas from SIRT1^STOP^ and SIRT1^ΔSTOP^. Proliferation index is expressed as the percent of Ki-67 stained adenoma cells (averaged for at least 10 adenomas per cohort). Mitotic rate is calculated as the number of histologically identifiable mitotic figures per 10 high-power fields (400×). Values in B and C are means±s.d.

APC^min/+^, SIRT1^STOP^ control mice that did not overexpress SIRT1 (referred to as SIRT1^STOP^) showed the typical signs of tumor morbidity at 16 weeks of age, as evidenced by overt anemia and cachexia, whereas APC^min/+^ mice overexpressing SIRT1 (SIRT1^ΔSTOP^) displayed no overt signs of tumor associated morbidity (Fig. S1A, B). Examination of the gut lining at four months of age showed that the SIRT1^ΔSTOP^ transgenic mice had significantly smaller and fewer tumors along the intestinal tract (Fig. 2A). Quantification of the tumor burden revealed a 3 to 4-fold reduction in the number and size of adenomas within the small intestine and colon of the SIRT1^ΔSTOP^ mice (Fig. 2B). Ki-67 is a granular component of the nucleolus that is expressed exclusively in proliferating cells and is used as a prognostic marker in human neoplasias. Adenomas of the SIRT1^ΔSTOP^ mice had a significant reduction in the numbers of mitoses (per high-power field) and Ki-67 staining, demonstrating that there was a decrease in adenoma proliferation (Fig. 2C). These data demonstrate that overexpression of SIRT1 in the gut at similar levels to those induced by CR is sufficient to mimic the tumor suppressive effect of CR in the APC^min/+^ mouse.

**Figure 3:**
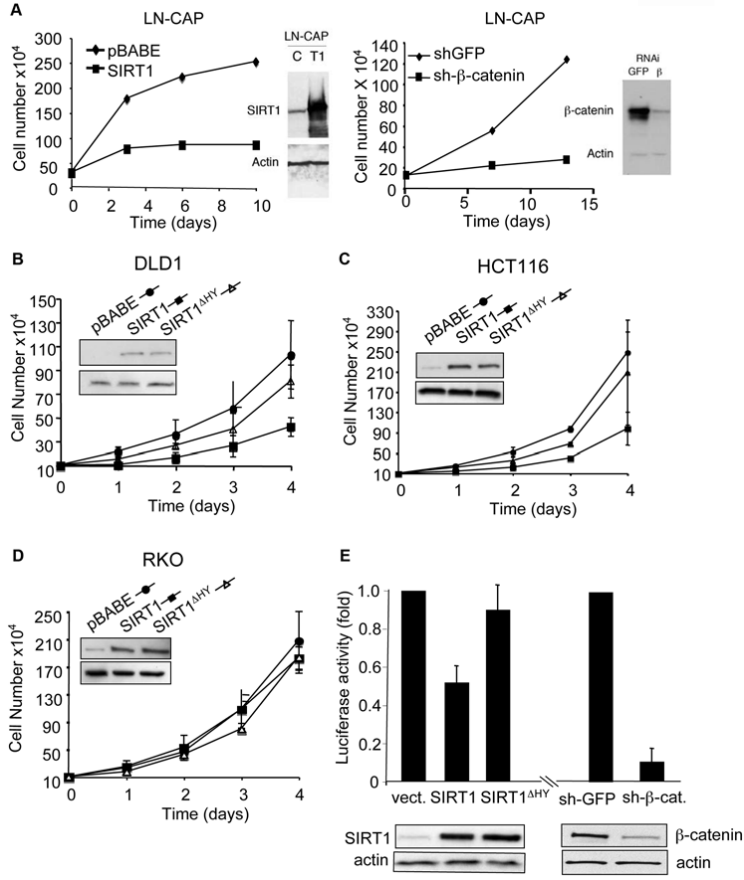
SIRT1 inhibits β-catenin driven cell proliferation and transcriptional activity. (A–D) Stable LN-CAP, DLD1, HCT116 and RKO cell lines expressing the indicated product were seeded and cell number was monitored at different time points. Western blot were performed with SIRT1, actin or β-catenin antibodies. (E) DLD1 stable cell lines expressing Topflash-LuciferasePEST were infected with the indicated constructs. Cells were analyzed by western blot with antibodies against SIRT1 and β-catenin. Luciferase activity was normalized for total sample protein and represents three independent experiments done in quadruplicate.

To gain insights into the mechanisms by which SIRT1 reduces cellular proliferation, we examined the effect of SIRT1 on the growth rate of several well characterized cancer cell lines. The proliferation of LNCaP prostate cancer cells was greatly reduced by overexpression of SIRT1 and the effect was similar to suppressing β-catenin itself (Fig. 3A). This observation suggested that SIRT1 and β-catenin function in the same cellular context. To further explore this possibility, we overexpressed SIRT1 and a catalytically inactive SIRT1 mutant (SIRT1^ΔHY^) in different human colon cancer cell lines whose growth is driven by constitutively active β-catenin (HCT116 and DLD1). A human colon cancer cell line in which β-catenin is inactive (RKO) served as a negative control. Increased SIRT1 expression greatly reduced proliferation in both colon cancer cell lines with constitutively active β-catenin but not in the β-catenin-inactive cell line (Fig. 3A–D). The SIRT1^ΔHY^ catalytic mutant had no significant effect on cellular proliferation in any of the cell lines (Fig. 3B–D). Thus, SIRT1 suppresses β-catenin-driven proliferation and its catalytic activity is apparently required for this effect.

To further understand the mechanism by which SIRT1 suppresses β-catenin-driven proliferation, we engineered the DLD1 cell line to contain a stably integrated reporter with β-catenin response elements (Super8XTopflash-Luciferase^PEST^). Knockdown of β-catenin dramatically reduced reporter activity, demonstrating the dependence of the reporter on endogenous β-catenin activity (Fig. 3E). Overexpression of SIRT1 reduced reporter activity by ∼2-fold, whereas the SIRT1^ΔHY^ catalytic mutant had no effect (Fig. 3E), suggesting that the anti-proliferative effects of SIRT1 are mediated by its ability to suppress the transcriptional activity of endogenous β−catenin and that this requires SIRT1 deacetylase activity.

**Figure 4:**
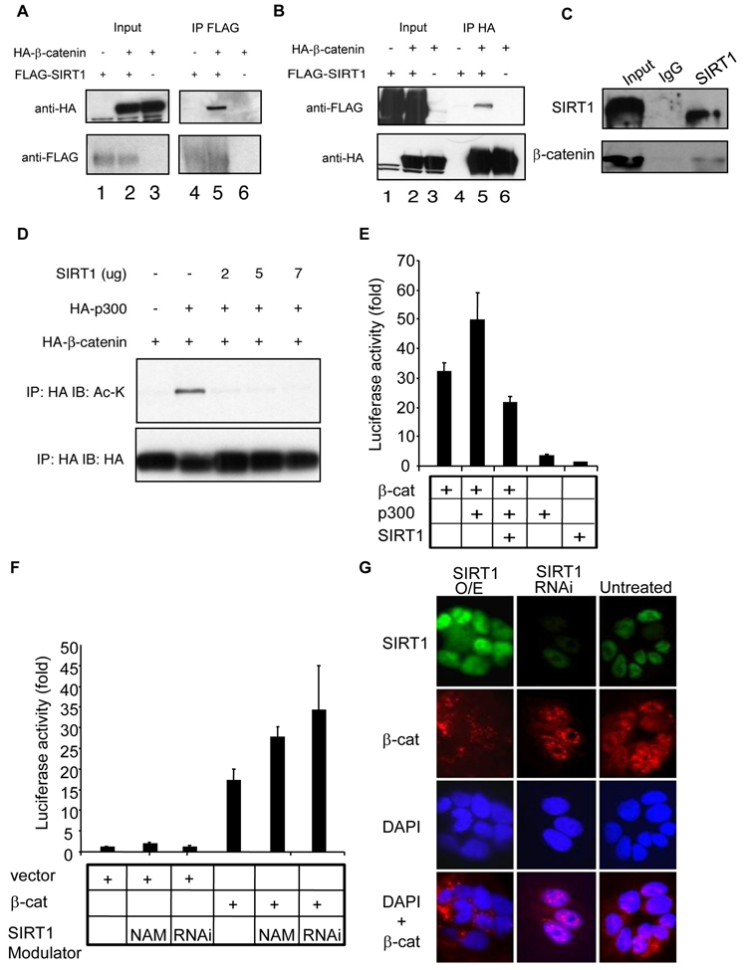
SIRT1 represses β-catenin transcriptional activity by directly interacting with and deacetylating β-catenin. (A) Human 293T cells were transiently transfected with HA-S33Y-β-catenin in combination with either FLAG-tagged SIRT1 or vector control. Aliquots of total protein were subjected to immunoprecipitation with anti-FLAG antibody (IP FLAG). Immunoprecipitated proteins were immunoblotted with anti-HA (upper panel) and anti-FLAG (lower panel). Left lanes, unprocessed extracts (input). (B) Human 293T cells were transfected as in panel A. Proteins immunoprecipitated with anti-HA antibody and immunoblotted with anti-FLAG (upper panel), and anti-HA (lower panel). Left lanes, unprocessed extracts (input). (C) Immunoprecipitation of SIRT1 from LN-CAP cell extracts using anti-SIRT1 antibody or normal rabbit IgG as a control (IgG). 10% of the immunoprecipitated protein was then blotted with anti-SIRT1 (upper panel) while the remaining 90% was blotted with anti-β-catenin antibodies. (D) 293T cells were transfected as indicated and lysed 48 hr later. Comparable levels of β-catenin were immunoprecipitated and blotted for acetylated-lysine residues (IP: HA IB: Ac-K; upper panel). The blot was reprobed for HA to demonstrate approximately equal levels of the HA-β-catenin (IP: HA IB: HA; lower panel). (E–F) 293T cells were transfected as indicated together with the TOP-FLASH luciferase and PRL-TK Renilla luciferase construct. Nicotinamide (NAM) or retroviral SIRT1 shRNA (RNAi) was added as indicated. Data are normalized with respect to Renilla luciferase activity. The data are means±s.d. from samples performed in triplicate. (G) Indirect immunofluorescence staining of DLD-1 colon cancer cells infected with empty retrovirus or virus containing SIRT1 shRNA (RNAi) or SIRT1 cDNA (overexpression, O/E). Percent of cells with high, medium, or low levels of nuclear β-catenin staining for untreated: 6.5, 80.6, 12.9; SIRT1 O/E: 0, 29.4, 70.5; SIRT1 RNAi: 60.0, 32.0, 0.8. Images were taken at 100× magnification.

Acetylation of β-catenin by p300/CBP potentiates its oncogenicity by increasing β-catenin/TCF avidity at target gene promoters [26]. To test whether SIRT1 modifies β-catenin, HEK293T cells were transfected with a mutant form of β-catenin that constitutively localizes to the nucleus (S33Y-β-catenin) [27]. In these cells SIRT1 co-immunoprecipitated with β-catenin (Fig. 4A) and vice versa (Fig. 4B). An interaction between endogenous SIRT1 and β-catenin was also apparent (Fig. 4C).

To test whether SIRT1 inhibits β-catenin by modulating its acetylation, we transfected 293T cells with S33Y-β-catenin, the acetyltransferase p300, and increasing amounts of SIRT1. We found that p300 acetylated β-catenin (Fig. 4D) and potentiated its transcriptional activity, as has been previously reported [27] (Fig. 4E). The addition of SIRT1, however, abolished the acetylated form of β-catenin and significantly diminished β-catenin activity when it was co-transfected with p300 (Fig. 4D, E). Conversely, treating cells with the SIRT1 inhibitor nicotinamide (NAM) [28] or suppressing SIRT1 with a retroviral shRNA vector (Fig. 4F) increased luciferase reporter activity. These data show that SIRT1 promotes the deacetylation of β-catenin, thereby reducing its ability to act as a trans-activator.

The constitutive presence of β-catenin in the nucleus is associated with its oncogenic function and clinically with a poor patient prognosis [29]. To test whether SIRT1 might repress β-catenin by altering its localization, immunofluorescence was performed on cells transfected with shRNA or overexpression constructs for SIRT1. Suppression of SIRT1 in the DLD1 colon cancer cells increased the amount of nuclear β−catenin while overexpression of SIRT1 in the same cell line led to a dramatic reduction in the nuclear β-catenin pool (Fig. 4G).

**Figure 5:**
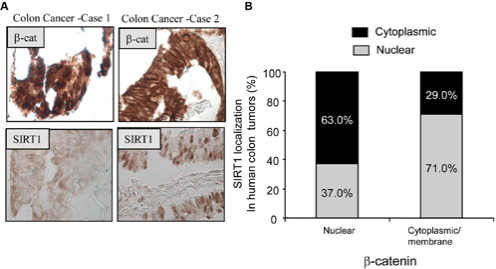
SIRT1 expression occurs in a subset of human colon cancers and inversely correlates with the nuclear localization of β-catenin. (A) Representative images illustrating SIRT1 and β-catenin subcellular expression in human colon tumors. For each colon cancer case shown a text box insert indicates the detected protein (Image magnification 200×). (B) Correlation of SIRT1 and β-catenin expression in human colon tumors. The bar graph depicts cumulative immunostaining data from a tissue microarray of 81 colon cancer cases. Nuclear expression was scored as either no expression, weak expression, or moderate/strong expression. Positivity in nucleus was defined as moderate/strong expression. All slides were interpreted by two board certified pathologist blinded from any other clinical and laboratory data.

To analyze the clinical relevance of our findings, we analyzed SIRT1 and β-catenin subcellular expression in a tissue microarray containing 81 human colon cancer samples. We found that a subset of colon cancers express SIRT1 in the nucleus (47/81 cases; 58%). When β-catenin expression was scored in these same colon cancers, a highly significant inverse correlation between the level of SIRT1 expression and nuclear β-catenin localization became apparent (p≤0.003, odds ratio 0.24 with 95% confidence interval 0.093–0.63) (Fig. 5A, B). Collectively, these observations suggest that modulation of β-catenin subcellular localization is an important component of the anti-tumorigenic effects of SIRT1 with potential diagnostic and therapeutic clinical relevance.

## Discussion

Here we describe a physiologically relevant tumor suppressive role for SIRT1 in colon cancer formation and growth. We observed that SIRT1 expression in the normal intestine occurs specifically in the enterocytes, the precursor cells that undergo neoplastic transformation in colon cancers and that SIRT1 is upregulated in rodent intestines in response to CR. We show that overexpression of SIRT1 reduces proliferation in colon cancer cell lines and that overexpressing SIRT1 in the enterocytes of APC^min/+^ animals mimics the tumor suppressive effects of CR on this colon cancer model. These observations are consistent with a recent *in vitro* study that implicates SIRT1 as a nutrient sensitive growth suppressor [30]. While SIRT1 is expressed in our transgenic mice at higher levels than seen in the intestines of CR treated rodents (7 fold (SIRT1) versus 2 fold (CR)), this level of overexpression is, nonetheless, consistent with findings that SIRT1 can be physiologically upregulated 5–10 fold *in vivo* [31]. Since the tumor suppressive effects mediated by SIRT1 eclipse those seen by CR (70% reduction (SIRT1) versus 40% reduction (CR)) [16] we cannot exclude the possibility that SIRT1 also inhibits tumor growth by a CR-independent mechanism. Nevertheless, our data provide *in vivo* evidence that overexpression of SIRT1 at physiologically relevant levels, can suppress tumor formation and growth.

In this study, we also present evidence that SIRT1 interacts with and suppresses β-catenin, the transcription factor that drives tumors in the APC^min/+^ model and a variety of human tumors. We find that SIRT1 overexpression inhibits the growth of colon cancer cells dependent on β-catenin activity, suppresses the localization of β-catenin to the nucleus, and significantly attenuates its ability to activate transcription. These effects were not observed in the SIRT1-HY mutant demonstrating that SIRT1 deacetylase activity is required, and raising the possibility that SIRT1 directly targeted β-catenin for deacetylation.

Previous studies have shown that β-catenin is acetylated by p300/CBP and the acetylated form of the protein has increased transcriptional activity. This finding implies that the putative deacetylase that counteract p300/CBP would be useful as a cancer therapeutic target [32]. In this study, we identify SIRT1 as a deacetylase that antagonizes p300/CBP and deacetylates β-catenin, thus slowing cellular proliferation and tumor growth *in vivo*.

Together, our data sheds light on the ability of SIRT1 to inhibit β-catenin activity and provides mechanistic insight into the anti-tumorigenic effects of SIRT1 in a well characterized colon cancer model. Given that SIRT1 was discovered as a homolog of a longevity gene, it is interesting to note the growing evidence for a link between Wnt/β-catenin signaling and age-associated diseases. β-catenin has been linked to other age-associated malignancies such as melanoma, multiple myeloma, and prostate cancer. There is also the recent discovery that upregulation of Wnt/β-catenin signaling accelerates aging in the mouse [23], [24]. Thus, it will be worth investigating whether SIRT1 can provide protection against other age-associated diseases on account of its ability to suppress Wnt/β-catenin signaling.

In summary, using biochemistry, mouse genetics, and clinical tumor specimens we have found that SIRT1, a diet-responsive gene, is a regulator of β-catenin and has a tumor suppressive function. We conclude that mammalian longevity genes with anti-apoptotic functions, surprisingly do not necessarily lead to increased tumorigenesis. In fact, we find the opposite is likely the case for SIRT1. These studies begin to answer an important biological question regarding the function of a key longevity gene in cancer development and growth and suggest a previously unidentified therapeutic potential for SIRT1 activators in cancer.

## Materials and Methods

### Rodents

A Cre-inducible SIRT1 expression construct was generated in which a *loxP* flanked transcriptional STOP element was inserted between a CAGGS promoter and the SIRT1 cDNA. This construct was targeted into the mouse Collagen A1 locus using flp recombinase-mediated genomic integration as described previously (1). MES cells carrying a single copy of the SIRT1^STOP^ construct were identified by resistance to the antibiotic marker hygromycin and Southern blotting. PCR primers and construct maps are available upon request. Two clones were injected into blastocysts and both generated pups, ∼90% of which displayed germ-line transmission. Tumor bearing mice that were analyzed had been backcrossed at least four generations into C57/BL6. APC^min/+^ and Villin-Cre transgenic mice strains were obtained in the C57/BL6 background from Jackson Labs (Bar Harbor, ME). SirT1^STOP^ animals were backcrossed two generations into C57BL/6 mice before crossing to APC^min/+^ to generate SirT1^STOP^; APC^min/+^ double transgenics. These animals were bred to Villin-Cre transgenic mice to generate a cohort of SirT1^ΔSTOP^; Vil-Cre; APC^min/+^ animals. Animals were maintained at Harvard Medical School and experiments were approved by the Animal Care Committee of Harvard Medical School.

Male Fischer-344 (F344) rats were bred and reared in a vivarium at the Gerontology Research Center (GRC, Baltimore, MD). From weaning (2 Wks), the rats were housed individually in standard plastic cages with beta chip wood bedding. Control animals were fed a NIH-31 standard diet ad libitum (AL). At 1 month of age the calorie restricted (CR) animals were provided a vitamin and mineral fortified version of the same diet at a level of 40% less food (by weight) than AL rats consumed during the previous week. Filtered and acidified water was available ad libitum for both groups. The vivarium was maintained at a temperature of 25°C with relative humidity at 50% on a 12/12-hour light/dark cycle (lights on at 6:00 a.m.) All animals were 6 months of age and sacrificed between 9:00 and 11:00 a.m. The intestine was quickly removed and thoroughly flushed with ice cold PBS and placed into liquid nitrogen then stored at −80°C until processed for Western blotting using standard procedures.

### Animal Pathology, Histopathology and Immunohistochemical analysis

For gross tumor analysis, the entire intestine was excised immediately after sacrifice, opened lengthwise and washed with cold phosphate-buffered saline (PBS) while pinned down a solid support. Adenomas larger than 0.5mm from the proximal (10 cm distal to the pylorus), distal (10 cm proximal to the cecum), and middle (∼50% of total intestinal length) small intestine as well as the colon were scored. Intestines were prepared using the Swiss roll method by rotating them around a glass pipette tip. Tissues were fixed and embedded in paraffin using standard histology protocol. Precise tumor size was scored microscopically on hematoxylin/eosin stained of mouse intestines using a microscope with an eyepiece micrometer. Immunohistochemical analysis of rodent tissue was performed with rabbit anti-SIRT1 antibody (Upstate Biotechnology, cat #07-131), rabbit anti-β catenin (abcam #2982), mouse anti-β-catenin (Clone 14, BD transduction labs) and rat anti-mouse Ki-67 (Dako).

### Immunohistochemical and Statistical Analyses

Tissue microarrays (TMAs) were constructed as previously described [33] using the Automated Arrayer (Beecher Instruments, Sun Prairie, WI USA). For β-catenin and SIRT1 immunohistochemistry, antigen retrieval was performed; deparaffinized tissue sections were treated by a microwave for 15 min in citrate buffer (BioGenex, San Ramon, CA) in a pressure cooker. Tissue sections were incubated with 3% H_2_O_2_ (15 min) to block endogenous peroxidase, then incubated with 10% normal goat serum (Vector Laboratories, Burlingame, CA) in PBS (10 min), followed by 10 min incubation in serum free protein block (DAKO, Carpinteria, CA). Primary antibody against β-catenin (clone 14, BD Transduction Laboratories, Franklin Lakes, NJ) (dilution 1∶400) or SIRT1 (#1104; Epitomics) (dilution 1∶100) was applied for 1 hour at room temperature. Secondary antibody (BioGenex) (20 min), and then streptavidin peroxidase conjugate (BioGenex) were applied (20 min). Sections were visualized by diaminobenzidine (DAB) (2 min) and methyl-green counterstain. Nuclear expression was recorded as either no expression, weak expression, or moderate/strong expression. Positivity in nucleus was defined as moderate/strong expression. All β-catenin-stained TMA slides were interpreted by a pathologist (S.O.) blinded from any other clinical and laboratory data. All SIRT1-stained TMA slides were interpreted by a pathologist (R.F.) blinded from any other clinical and laboratory data. For statistical analysis, chi-square test was performed for categorical data using the SAS software program (Version 9.1, SAS Institute, Cary, NC). The p-value was two-sided, and statistical significance was set at p ≤ 0.05.

### Plasmids

pcDNA3-FLAG-SIRT1, pBABE-Puro-hSir2 (SIRT1), pBABE-Puro-SIRT1^ΔHY^, pcDNA-HA-S33Y-β-catenin and pBABE-Puro-S33Y- β-catenin have been described before. SIRT1 RNAi plasmids were constructed by cloning the sequences into the pSUPER.retro plasmid (oligoEngine, Seattle, WA). One TOPFLASH plasmid was purchased from Upstate Biotechnology (Lake Placid, NY) while the second was generated by cloning the tandem TCF binding sites and TA-promoter from SUPER8xTOPFLASH (kind gift of Randall Moon) into the Luciferase-PEST plasmid pGL4.15 (Promega, Madison, WI).

### Cell Transfections, Infections and Immunocytochemistry

293T, LN-CAP, DLD1, HCT116 and RKO cells were maintained in the recommended tissue culture media (American Type Culture Collection (ATCC), Manassas, VA) and grown in a humidified incubator containing CO_2_ (5% v/v) at 37°C. For over-expression experiments, plasmids were transfected by the Fugene 6 method (Roche). For stable cell line generation, DLD1 cells were selected in hygromycin for two weeks and single colonies were picked and expanded. For retroviral production, 293T cells were transfected with the overexpression or RNAi plasmids simultaneously with packaging plasmids gag-pol and VSV-G or pCL-ampho. The media containing the progeny virus released for the 293T cells was collected and used to infect the cells for 3–6 hours in the presence of 8 µg/ml polybrene (Sigma Aldrich, St. Louis, MO). The medium was changed and cells were incubated for additional 24–48 hour incubation. They were selected with puromycin (Sigma Aldrich) for 24–48 hours and then trypsinized and seeded for experiments.

For immunocytochemistry, cells were plated on 8 mm six well Teflon printed slides (Electron Microscopy Sciences). Twenty four hours after plating, cells were fixed by incubation with 4% Paraformaldehyde for 30 minutes. Fixed cells were then incubated with primary antibodies to SIRT1 (#1104; Epitomics (dilution 1∶100) and active β-catenin (05-665; Chemicon) (dilution 1∶100) for 2 hours followed by incubation with secondary antibodies (Alexa Fluor 488 goat anti-rabbit IgG and Alexa Fluor 568 goat anti-mouse IgG; Molecular Probes) (dilution 1∶400). Cells were incubated with 10 µg/ml Hoechst, washed and mounted with a coverslip. At least 20 cells were scored per experiment and image capture was performed using an Olympus BX50 microscope.

### Protein Extraction and Immunoprecipitation

Cell extracts analyzed directly by Western blotting were prepared by cell lysis in 1× SDS loading buffer followed by boiling and Western analysis. Cell extracts for immunoprecipitation were prepared by resuspending phosphate-buffered saline-washed cell pellets in 1 ml of Nonidet P-40 (NP-40) extraction buffer (50 mM Tris-HCl [pH 8.0], 150 mM NaCl, and 1% Nonidet P-40) supplemented with EDTA-free protease inhibitor cocktail tablets (Roche) with 10 mM Nicotinamide (NAA) and 5 µM Trichostatin-A (TSA). Following incubation on ice for 30 minutes, non-extractable material was removed by centrifugation at 17,000 *g* for 10 min at 4°C, and the cleared supernatants were employed for immunoprecipitation. Lysates were immunoprecipitated (2 hr), washed 4 times with NP-40 buffer and were processed by Western blotting.

### Proliferation Assays

DLD1, HCT116 and RKO cell lines infected with the appropriate construct were seeded in 24 well plates at a density of 10,000 cells. Cells were trypsinized and analyzed by Coulter counting at given time points.

### Luciferase Assay

Luciferase assay was done as instructed by the dual luciferase reporter assay system or firefly luciferase assay system (Promega, Madison, WI).

### Western Blotting and RNA Analysis

For expression studies, animal intestines were flushed with cold PBS and either homogenized whole or scraped to enrich for enterocytes. Protein extracts were prepared by dounce homogenization in standard lysis buffer, subjected to SDS-PAGE and transferred onto PVDF membranes. Membranes were immunoblotted using rabbit polyclonal antibody to SIRT1 (Upstate Biotechnology, cat #07-131) and rabbit polyclonal antibody to β-actin (Abcam cat #8226). Densitometric analysis was performed on scanned images of blots using ImageJ software (NIH Image analysis website http://rsb.info.nih.gov/ij/)

## Supporting Information

Figure S1 APC^min/+^ mice overexpressing SIRT1 in gut show less signs of morbidity. (A) APC^min/+^ mice overexpressing SIRT1 (SIRT1^ΔSTOP^) show less anemia than their non SIRT1 overexpressing age matched counterparts (SIRT1^STOP^) as reflected by the color of their paws. (B) Graph depicting average body weight of mice at time of sacrifice (n = at least 4 per group). Error bars represent average±S.D.
(2.31 MB TIF)
