# Correction: The SIRT1 Deacetylase Suppresses Intestinal Tumorigenesis and Colon Cancer Growth

**DOI:** 10.1371/journal.pone.0305277

**Published:** 2024-06-06

**Authors:** Ron Firestein, Gil Blander, Shaday Michan, Philipp Oberdoerffer, Shuji Ogino, Jennifer Campbell, Anupama Bhimavarapu, Sandra Luikenhuis, Rafael de Cabo, Charles Fuchs, William C. Hahn, Leonard P. Guarente, David A. Sinclair

After publication of this article [1], concerns were raised about Fig 1. Specifically, in Fig 1C, a background area below the band in the WT lane appears similar to a background region in the second FRT/+ lane.

**Fig 1:**
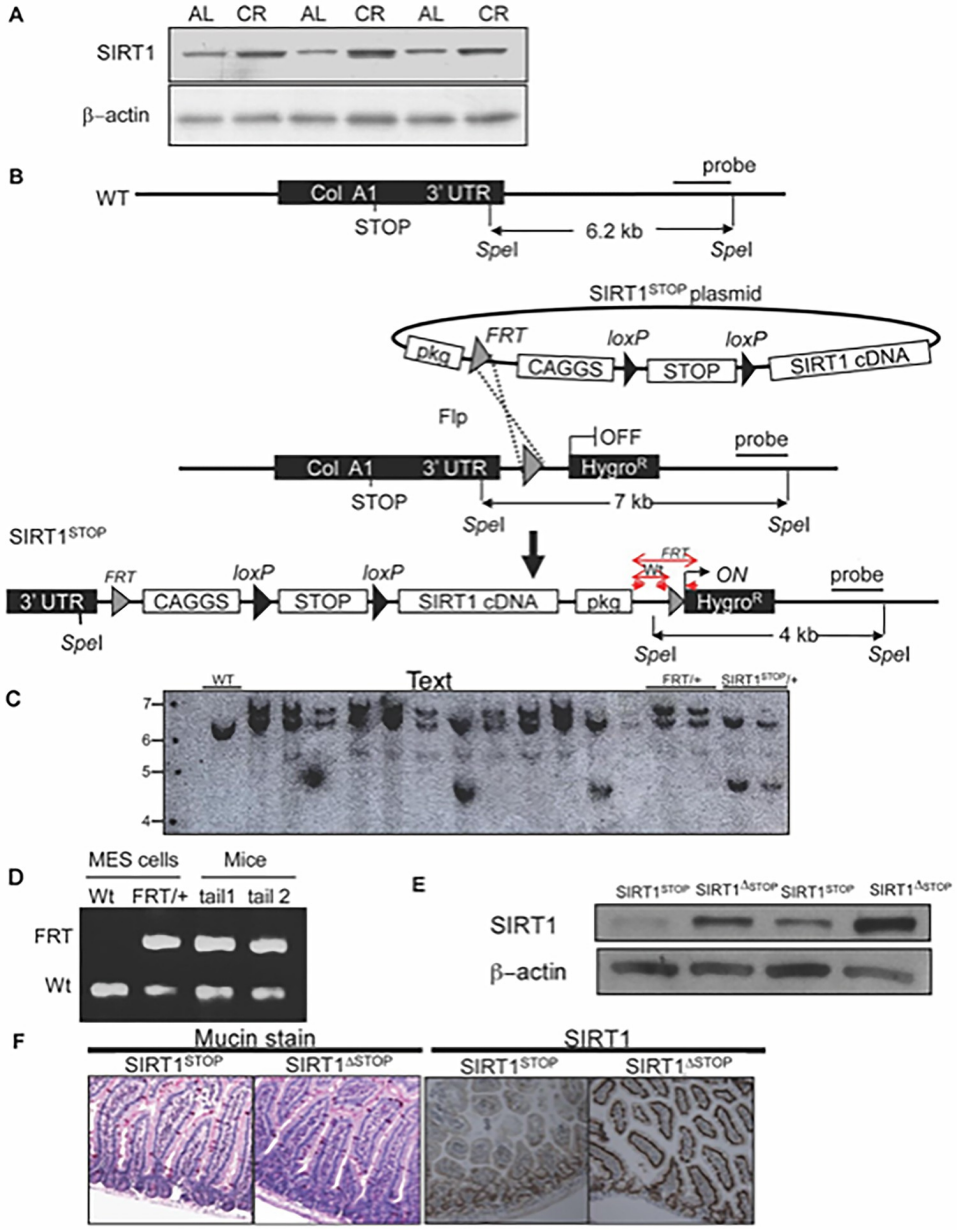
Generation of the conditional SIRT1 transgenic mice that mimic calorie restriction induced SIRT1 overexpression. (A) Western blot analysis showing expression levels in the gut epithelium of SIRT1 in ad libitum-fed (AL) or calorie restricted (CR) rats. β-actin served as the loading control in all lanes. (B) Schematic representation of the strategy used for the generation of the floxed SIRT1 mouse embryonic stem (MES) cells. SIRT1 was cloned downstream of a constitutive CAGGS promoter followed by a transcriptional loxP-STOP-loxP cassette. This construct was specifically targeted in the 3′ UTR of the collagen A1 locus (ColA1) of mouse embryonic stem cells (MES) cells by FLP recombination. The targeted MES cells were injected into blastocysts. Red arrows indicate location of the SIRT1-Tg genotyping primers. (C) Southern blot showing the confirmation of the SIRT1^STOP^ single integration into the Col1A locus of MES cells. (D) PCR confirming the germline transmission of the SIRT1^STOP^ transgene to the chimaeras’ offspring. (E) Western blot showing the levels of SIRT1 in the triple transgenic mice overexpressing SIRT1 (SIRT1Δ^STOP^) and controls (SIRT1^STOP^). β-actin served as the loading control in all lanes. (F) Mucin stain and immunohistochemistry of SIRT1 in the small intestine of experimental (SIRT1Δ^STOP^) and controls (SIRT1^STOP^) animals.

The corresponding author provided the original, uncropped image and a corrected Fig 1C. They noted that the original Southern blot image pertaining to Fig 1C has no region of similarity. Please see the correct Fig 1 here.

The raw data underlying the corrected figure are provided in S1 File. The image data did not clarify the reason for background region similarities in the published figure, but the *PLOS ONE* Editors are satisfied that the raw data support the published results and the principal bands appear similar in the published figure and data file.

It was also noted that Fig 1C and 1D are not cited in the article’s text and there are reporting errors in sentence 6 of the published Results section. The sixth sentence of the first paragraph of the Results section is hereby replaced with the following:

We confirmed single integration of the SIRT1^STOP^ construct into the collagen A1 locus (Col1A) of mouse embryonic stem cells (MES) by Southern blotting (Fig 1C) and performed PCR to track the transmission of the SIRT1^STOP^/+ transgene to the chimeras’ offspring (Fig 1D). The SIRT1 protein levels in the gut of SIRT1^ΔSTOP^ mice were increased approximately 7-fold (Fig 1E) compared to SIRT1^STOP^ controls, and the morphology of villi appeared otherwise normal (Fig 1F).

The authors apologize for the errors in the published article.

## Supporting information

S1 File Raw image data underlying Fig 1C. (TIFF)
