# Supplementary figures and images for: The SIRT1 Deacetylase Suppresses Intestinal Tumorigenesis and Colon Cancer Growth
Source: PLoS One. 2008 Apr 16;3(4):e2020. doi: 10.1371/journal.pone.0002020 (PMC2289879; doi:10.1371/journal.pone.0002020)

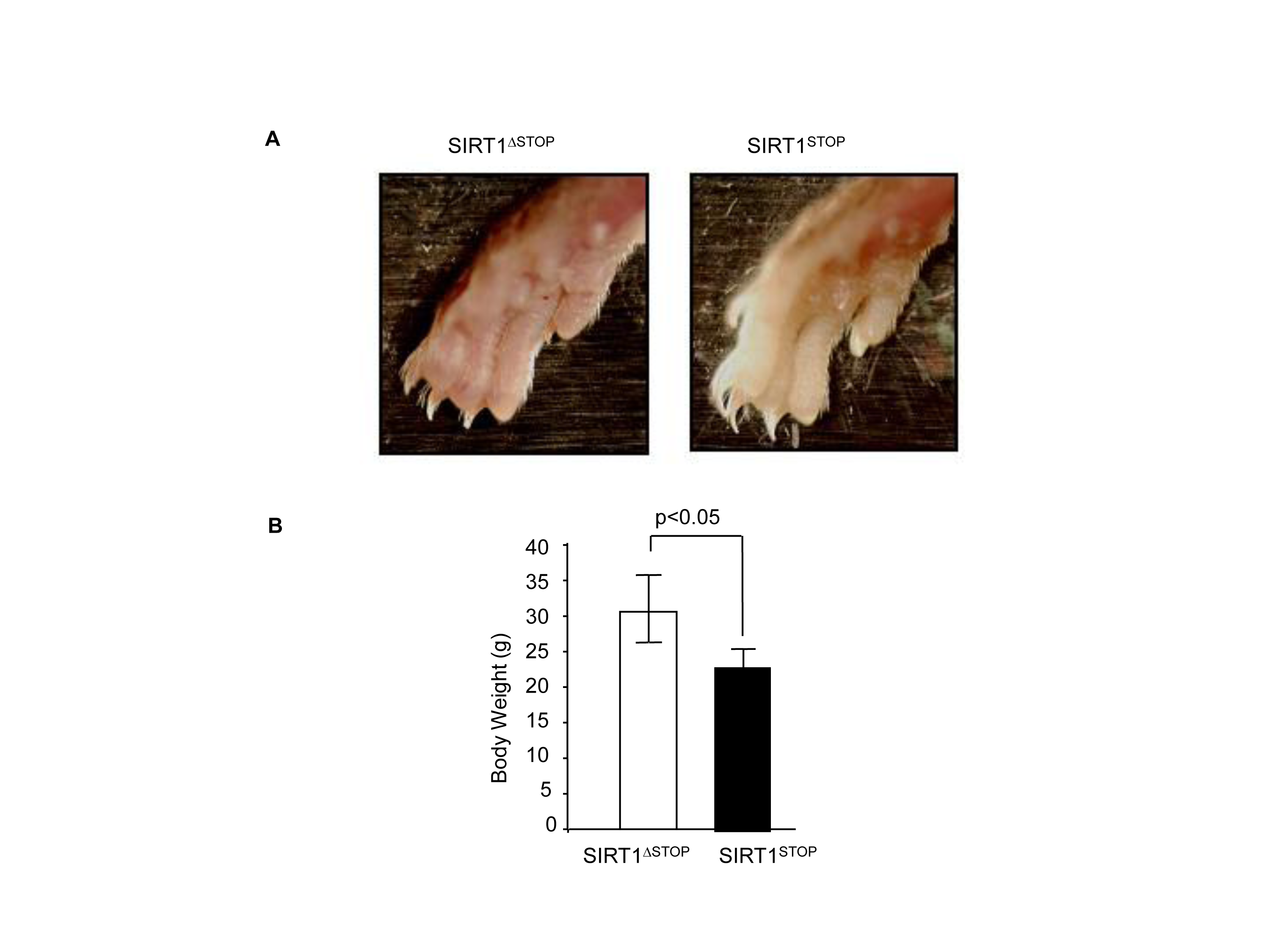

Supplement: Figure S1 — APCmin/+ mice overexpressing SIRT1 in gut show less signs of morbidity. (A) APCmin/+ mice overexpressing SIRT1 (SIRT1ΔSTOP) show less anemia than their non SIRT1 overexpressing age matched counterparts (SIRT1STOP) as reflected by the color of their paws. (B) Graph depicting average body weight of mice at time of sacrifice (n = at least 4 per group). Error bars represent average±S.D. (2.31 MB TIF) [file pone.0002020.s001.tif]
