# Supplementary figures and images for: Correction: The SIRT1 Deacetylase Suppresses Intestinal Tumorigenesis and Colon Cancer Growth
Source: PLoS One. 2024 Jun 6;19(6):e0305277. doi: 10.1371/journal.pone.0305277 (PMC11156279; doi:10.1371/journal.pone.0305277)

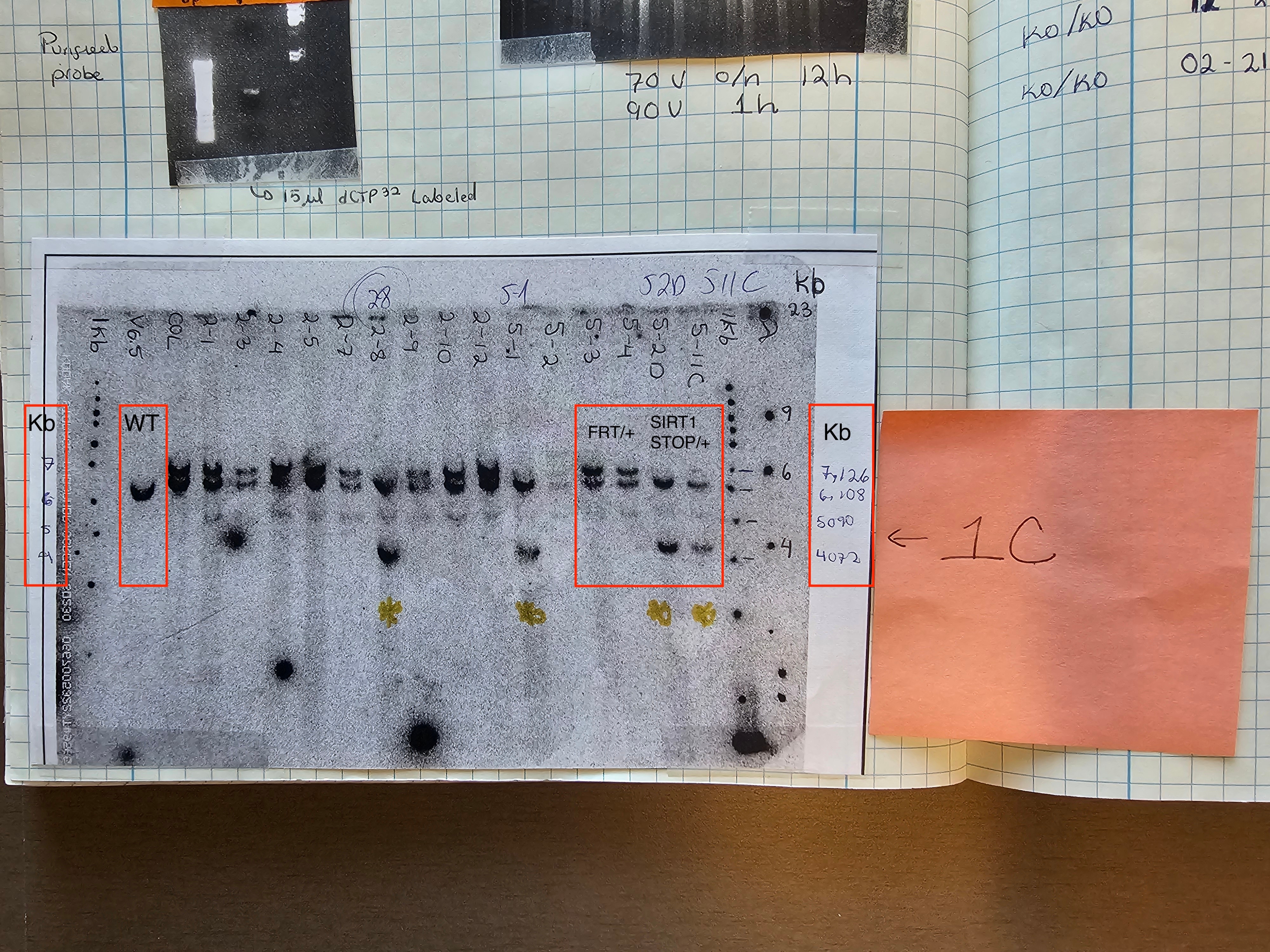

Supplement: S1 File — (TIFF) [file pone.0305277.s001.tiff]
